# Identifying Clusters of Health Behaviors in a Japanese Working Population at Risk for Non-Communicable Diseases: A Latent Class Analysis of 12,168 Individuals

**DOI:** 10.1016/j.ssmph.2023.101539

**Published:** 2023-10-16

**Authors:** Takahiro Miki, Kojiro Yamamoto, Masashi Kanai, Kento Takeyama, Maki Iwatake, Yuta Hagiwara

**Affiliations:** a PREVENT Inc.; b College of Transdisciplinary Sciences for Innovation, Kanazawa University, Japan; c Graduate School of Rehabilitation Science, Saitama Prefectural University, Japan

**Keywords:** Noncommunicable diseases, Health behaviors, Latent class analysis, Japanese population, Clustering

## Abstract

**Introduction:**

Noncommunicable diseases (NCDs) have become a significant global problem. Health behaviors are associated with NCDs, and characterizing populations using a public health approach can help provide specific interventions according to their characteristics. This study aims to examine the formation of clusters of health behavior combinations in the Japanese working population at risk of NCDs, taking into account the influences of age and gender, using latent class analysis.

**Methods:**

Participants were individuals at risk for NCDs but had not previously been diagnosed with any. Latent class analysis (LCA) was used to study clustering based on basic characteristics and health behaviors. All statistical analyses were conducted using R (Version 4.0.4) and the “poLCA” package (Version 1.6.0).

**Results:**

This study included 12,168 participants. LCA compared models with one to six latent classes. The five-class model was determined to be the most appropriate based on Bayesian Information Criterion, Akaike Information Criterion, and G^2 values, as well as distinguishable cluster characteristics. Cluster 1: “having healthy lifestyles but disliking hospitals”; Cluster 2: “women with healthy lifestyle behaviors”; Cluster 3: “general population”; Cluster 4: “middle-aged group in need of lifestyle improvement”; Cluster 5: “a group receiving treatment for lifestyle-related diseases."

**Conclusions:**

This study reveals discernible health behavior patterns in a sample of the Japanese population using large real-world data, suggesting the effectiveness of distinct approaches when considering a population approach to public health.

## Highlights

• Latent class analysis reveals distinct health behavior clusters in Japanese working population.
• Five clusters identified, considering age, gender, and health behavior patterns.
• Findings may inform tailored public health interventions for noncommunicable disease prevention.

## 1 Introduction

Noncommunicable diseases (NCDs), commonly known as “lifestyle diseases,” such as diabetes, hypertension, and heart disease, have become more frequent in recent years and are a significant global problem (World Health Organization, 2022). It is estimated that approximately one billion people worldwide suffer from hypertension and that 60% of adults will suffer from hypertension by 2050 (Mills et al., 2020). The number of diabetics in the United States is projected to rise to 48.3 million by 2050 (Narayan et al., 2006). In India, 10% of adults suffer from hypertension, and 25 to 30 million people have diabetes. Three out of every 1000 people suffer from stroke. Heart attack deaths are projected to rise from 1.2 million to 2 million by 2010 (Sharma & Majumdar, 2009). The risk of stroke is also high in Japan (Pham et al., 2007). Heart disease is also the second most common cause of death among the Japanese population (Iso, 2011). The accumulation of poor health behaviors causes NCDs (Glanz et al., 1991). Health behaviors are health-related behavioral habits, such as frequency of alcohol consumption, smoking, and exercise. These poor health behaviors are associated with poor health status, including early mortality (GBD 2019 Risk Factors Collaborators, 2020; Loef & Walach, 2012). For example, it is widely known that smoking is a risk factor for coronary heart disease (Bazzano et al., 2003). The frequency of alcohol consumption affects the incidence of many diseases, including cancer, neuropsychiatric disorders, and many cardiovascular and gastrointestinal infections (Shield et al., 2013). Health behavior is a widely used proxy for proneness to NCDs (Fitzpatrick et al., 2015; Södergren et al., 2014). A combination of unhealthy habits, rather than a single habit, is a risk factor. Furthermore, the combinations often exhibit specific common patterns (Poortinga, 2007; Schuit et al., 2002). For example, cigarette smokers drink large amounts of alcohol (de Vries et al., 2008; Linardakis et al., 2013). Therefore, it is crucial for epidemiologists to investigate the formation of common patterns and clustering of combinations of health behaviors (Conry et al., 2011; Schneider et al., 2009). Especially in public health population approaches, it is more effective to understand the population's characteristics and provide specific interventions that meet those characteristics. Many recent studies have examined clustering based on health behavior for various countries' populations (Chou, 2008; Conry et al., 2011; Griffin et al., 2014; Kang et al., 2010; Schuit et al., 2002). For example, a study in Ireland reported that several clusters can be formed according to health behaviors and that such clusters are similar to those of other European populations (Conry et al., 2011). Despite some differences according to age and gender in the UK study, they formed a clustering with some consistent characteristics (Mawditt et al., 2016). However, these were relatively small sample sizes or studies focused on Europe and the United States. While some studies have explored the clustering of health behaviors in various populations, there remains a significant gap in understanding how these patterns manifest in the Japanese working population. This demographic, with its unique cultural and societal nuances, may exhibit distinct health behavior patterns that are pivotal for targeted interventions. Given the unique cultural and societal nuances of Japan, it's essential to understand how these patterns manifest in the Japanese working population. It is not appropriate to generalize these results to different countries, including Japan, since different countries have different cultures and lifestyles. Moreover, these studies may not have thoroughly analyzed the influences of age and gender. Considering age and gender in the analysis may lead to more accurate clustering results and help in formulating effective preventive measures and strategies for specific age groups and genders. In addition, study samples have included a broad general population, including healthy individuals or those who already have severe NCDs. Since a vital perspective concerning NCDs includes early prevention, it would be beneficial to examine the characteristics of multiple health behaviors in a working population at risk for NCDs before they become diagnosed (Ezzati & Riboli, 2012; Williams et al., 2018). From the above, more studies are needed to examine clusters based on health behavior characteristics in the Japanese population. In particular, although most work environments mandate regular annual health screening, no studies have been conducted on approaches to secondary prevention for those in the workforce who require treatment above the reference values. From a practical perspective, the findings of the study could inform public health initiatives and intervention strategies for the working population in Japan. For example, understanding specific clusters and patterns of health behaviour can provide insights for implementing more effective prevention measures and awareness-raising campaigns.

Therefore, this study aims to conduct clustering analysis by health behavior of Japanese people in the working population who are at risk of NCDs to understand the characteristics of each subgroup. The study employed latent class analysis (LCA) for clustering health behaviors. This approach allows for a more detailed capture of combinations and patterns of health behaviors, potentially offering insights and results that differ from previous studies.

## 2 Method

### 2.1 Study design and participants

This study was a cross-sectional study. Participants were selected based on data collected by PREVENT Inc. (Nagoya, Japan), which conducted medical data analyses. The data source comprised anonymized, processed receipt information and medical check-up data provided by health insurance associations in Japan contracted with PREVENT Inc. stored at the company. The study analyzed data from the administrative claims-based database that included information on individuals who underwent health examinations at least once and were under health insurance coverage between April 2013 and July 2021. The study included individuals who had not previously been diagnosed with an NCD and who met one of the following criteria for inclusion.

• Blood pressure: systolic blood pressure 160 mmHg or higher and/or diastolic blood pressure 100 mmHg or higher
• Lipid: Low-Density Lipoprotein (LDL) cholesterol level 180 mg/dL or higher and/or High-Density Lipoprotein (HDL) cholesterol level 30 mg/dL or lower and/or triglycerides 500 mg/dL or higher
• Blood glucose: fasting blood glucose 140 mg/dL or higher and/or Hemoglobin ba1c (Hba1c) 7.0% or higher

These reference values are based on the values recommended by the national government and various academic societies in Japan (Abola et al., 2020; Araki et al., 2020; Umemura et al., 2019).

### 2.2 Measurements of demographic characteristics

Participant demographics included age, gender, and BMI. In addition, triglycerides, HDL cholesterol, LDL cholesterol, fasting blood glucose, Hba1c, and blood pressure (systolic/diastolic) were included.

### 2.3 Measurements of health-related behaviors

The underlying latent variable in the study is health-related behavior. To assess health-related behaviors, participants provided information using a standard questionnaire prepared by the national government. This questionnaire was administered once a year as part of the mandatory health check-ups for those working in Japan. This questionnaire was choice-based, simple, neutral and a trusted tool widely used throughout the country. It ensured that participants' responses were based on personal experience and perceptions. The health behaviors described below were assessed using this questionnaire.

#### 2.3.1 Exercise habits

Exercise habits were ascertained by asking whether the respondents exercised at least twice a week for at least 30 min. The participants were asked to choose between “Yes” and “No.”

#### 2.3.2 Smoking

The participants were asked if they were current smokers. They were asked to choose between “Yes” and “No.”

#### 2.3.3 Alcohol consumption

For alcohol consumption, participants were asked to choose from the options “daily,” “some time,” and “rarely or cannot.”

#### 2.3.4 Healthy eating habits

Regarding healthy eating habits, those who ate dinner within 2 h of bedtime at least three times a week were asked to select “Yes,” and those who ate less than that were asked to select “No.”

#### 2.3.5 Willingness to improve lifestyle

Participants were asked to select “Yes” if they wanted to improve their lifestyle habits and “No” if they did not.

#### 2.3.6 Number of hospital visits within the last year

The participants were asked to select the number of visits to a hospital within the last year from “fewer than 3,” “3 to 5,” “6 to 8,” “9 to 11,” and “12 or more.”

#### 2.3.7 Habits of taking medications within the last year

The participants were asked to answer “Yes” or “No” to whether or not they had taken any medications within the last year.

#### 2.3.8 History of vascular diseases

The participants were asked to answer “Yes” or “No” to whether they had a history of vascular disease.

### 2.4 Statistical analysis

Descriptive statistical analysis was conducted to describe and interpret the different classes. This study was analyzed using LCA to identify clusters based on the participants’ age, gender and health behaviors. LCA is a statistical method for finding latent classes of related cases from multivariate categorical data. LCA differs from cluster analysis and factor analysis because it allows for the inclusion of discrete and dichotomous variables (Weller et al., 2020). It is an appropriate method when variables are categorical rather than continuous. Furthermore, this method is flexible, and the choice of cluster criteria is less arbitrary (Hagenaars & McCutcheon, 2002).

LCA is considered more informative for describing health behaviors (Laska et al., 2009). This is because, according to one study, it is more appropriate for analyzing health behaviors as categorical variables (Ingledew et al., 1995). Concerning variable selection, in addition to health behaviors, age and gender were added to the variables in the study because they are widely known to be important factors (Meader et al., 2016).

The model selection criteria were based on the Bayesian Information Criterion (BIC), Akaike Information Criterion (AIC), and G^2 (Wang et al., 2017). For AIC and BIC, smaller values indicate a better fit of the model. BIC is the most reliable fit statistic and should be routinely reported (Nylund et al., 2007; Tein et al., 2013). Another study reported that the preferred model minimizes BIC and/or AIC values (Linzer & Lewis, 2011). The goal is also to select a model that minimizes G^2 without estimating an excessive number of parameters (Linzer & Lewis, 2011). In addition to the statistical criteria, the selection of the number of clusters was heavily influenced by the practical interpretability of the model. It was essential not only to have a model that fit the data well but also one that could be meaningfully interpreted and applied in real-world scenarios (Nathan, 2013). The true value of a model lay in its ability to be understood and utilized effectively. Therefore, while evaluating different models, special emphasis was placed on ensuring that the clusters formed were distinct, meaningful, and could provide actionable insights for health interventions. All statistical analyses were conducted using R (Version 4.0.4). The LCA was performed using the “poLCA” package (Version 1.6.0). Under the assumption that missing data were missing at random, the likelihood was calculated, including missing data based on the previous study (Linzer & Lewis, 2011).

## 3 Results

### 3.1 Participant characteristics

This study involved a total of 12,168 participants. The majority of participants were male, accounting for 80% (9703) of the total, with females comprising 20% (2465). The age distribution was concentration in the 40–59 age group, with 39% (4726) of the participants in the 40–49 age group and another 39% (4784) in the 50–59 age group. In terms of health behaviors, 78% (9098) of participants reported no regular exercise and 62% (7229) did not have healthy eating habits. Alcohol consumption varied, with 28% (3301) drinking alcohol every day and 38% (4450) saying they rarely or never drink alcohol. Smoking was reported by 32% (3801) of the participants. In terms of medical history and healthcare use, 95% (11,601) of participants reported not taking any medication in the past year and 95% (11,511) had no history of vascular disease. The majority had fewer hospital visits. 42% (5076) reported no hospital visits in the past year. The characteristics of the study participants were summarized in more detail in Table 1.

**Table 1:** The characteristics of the participants in the study.

Variables	Overall	Cluster 1	Cluster 2	Cluster 3	Cluster 4	Cluster 5
Total	12,168	2389	2215	4395	2098	1071
sex						
man	9703 (80%)	2348 (98%)	27 (1.2%)	4350 (99%)	2031 (97%)	947 (88%)
Woman	2465 (20%)	41 (1.7%)	2188 (99%)	45 (1.0%)	67 (3.2%)	124 (12%)
BMI	25.0 (3.9)	24.6 (3.3)	23.9 (4.2)	25.9 (4.0)	24.6 (3.4)	25.5 (3.8)
age						
<30	186 (1.5%)	0 (0%)	42 (1.9%)	126 (2.9%)	15 (0.7%)	3 (0.3%)
30∼39	1098 (9.0%)	0 (0%)	152 (6.9%)	725 (16%)	201 (9.6%)	20 (1.9%)
40∼49	4726 (39%)	21 (0.9%)	636 (29%)	2685 (61%)	1110 (53%)	274 (26%)
50∼59	4784 (39%)	1508 (63%)	1117 (50%)	859 (20%)	737 (35%)	563 (53%)
60over	1374 (11%)	860 (36%)	268 (12%)	0 (0%)	35 (1.7%)	211 (20%)
Neutral fat	185 (197)	174 (183)	120 (95)	193 (195)	238 (265)	201 (192)
(missing)	1	0	0	1	0	0
HDL	58 (17)	58 (17)	68 (18)	52 (15)	60 (17)	57 (17)
(missing)	3	0	1	2	0	0
LDL	157 (44)	147 (43)	173 (38)	163 (42)	144 (46)	151 (45)
(missing)	1	1	0	0	0	0
LDL/HDL ration	2.91 (1.04)	2.71 (1.03)	2.70 (0.90)	3.31 (1.01)	2.54 (0.97)	2.85 (1.03)
(missing)	4	1	1	2	0	0
Fasting blood glucose	106 (29)	115 (35)	99 (22)	105 (29)	105 (26)	110 (28)
(missing)	1154	207	211	421	221	94
Hba1c	5.86 (1.09)	6.10 (1.12)	5.77 (0.94)	5.85 (1.01)	5.69 (1.39)	5.93 (0.87)
(missing)	1004	217	188	322	166	111
SBP	133 (21)	138 (21)	129 (25)	130 (19)	138 (20)	134 (19)
DBP	85 (15)	87 (14)	79 (16)	84 (15)	90 (15)	86 (14)
exercise habits						
no	9098 (78%)	1452 (64%)	1749 (82%)	3499 (83%)	1616 (78%)	782 (75%)
yes	2627 (22%)	816 (36%)	377 (18%)	717 (17%)	460 (22%)	257 (25%)
(missing)	5557	1219	1112	1929	751	546
Healthy eating habits						
no	7229 (62%)	2030 (90%)	1773 (84%)	2121 (50%)	632 (30%)	673 (65%)
yes	4460 (38%)	225 (10.0%)	348 (16%)	2085 (50%)	1441 (70%)	361 (35%)
(missing)	479	134	94	189	25	37
Alcohol consumption						
everyday	3301 (28%)	794 (35%)	90 (4.2%)	0 (0%)	2083 (100%)	334 (32%)
sometimes	4017 (34%)	799 (35%)	596 (28%)	2275 (54%)	0 (0%)	347 (33%)
rarely, cannot	4450 (38%)	675 (30%)	1450 (68%)	1963 (46%)	0 (0%)	362 (35%)
(missing)	400	121	79	157	15	28
Willingness to improve lifestyle						
no	2729 (23%)	629 (28%)	311 (15%)	952 (23%)	637 (31%)	200 (19%)
yes	9010 (77%)	1643 (72%)	1818 (85%)	3269 (77%)	1441 (69%)	839 (81%)
(missing)	429	117	86	174	20	32
Smoking						
no	8171 (68%)	1600 (68%)	2083 (95%)	2646 (62%)	1066 (51%)	776 (73%)
yes	3801 (32%)	737 (32%)	103 (4.7%)	1652 (38%)	1026 (49%)	283 (27%)
(missing)	196	52	29	97	6	12
Number of hospital visits within the last year						
0	5076 (42%)	1761 (74%)	775 (35%)	1749 (40%)	791 (38%)	0 (0%)
≤ 3	3977 (33%)	464 (19%)	692 (31%)	1804 (41%)	953 (45%)	64 (6.0%)
≤ 6	1641 (13%)	164 (6.9%)	432 (20%)	559 (13%)	257 (12%)	229 (21%)
≤ 9	780 (6.4%)	0 (0%)	190 (8.6%)	153 (3.5%)	81 (3.9%)	356 (33%)
≤ 12	694 (5.7%)	0 (0%)	126 (5.7%)	130 (3.0%)	16 (0.8%)	422 (39%)
Habits of taking medications within the last year						
no	11,601 (95%)	2389 (100%)	2202 (99%)	4395 (100%)	2098 (100%)	517 (48%)
yes	567 (4.7%)	0 (0%)	13 (0.6%)	0 (0%)	0 (0%)	554 (52%)
History of vascular disease						
no	11,511 (95%)	2371 (99%)	2094 (95%)	4275 (97%)	2018 (96%)	753 (70%)
yes	657 (5.4%)	18 (0.8%)	121 (5.5%)	120 (2.7%)	80 (3.8%)	318 (30%)

^1^n (%) or Mean (SD).

Abbrevation: BMI, body mass index; HDL, high-density lipoprotein; LDL, low-density lipoprotein; SBP, systolic blood pressure; DBP, diastolic blood pressure.

### 3.2 Model selection of latent class analysis

The study compared models with one to six latent classes. The results of this study are provided in Table 2. Both the 5-class and 6-class models presented lower values for BIC, AIC, and G^2, suggesting their potential suitability. However, model selection is not solely based on statistical criteria; the interpretability and meaningfulness of the classes are equally crucial. The 5-class model yielded more distinct and interpretable groupings in terms of health behavior characteristics. In contrast, the 6-class model, despite its statistical fit, produced classes with overlapping or less discernible characteristics. Thus, prioritizing both statistical robustness and interpretability, the 5-class model was selected as it more clearly distinguishes between the characteristics of the groups and offers a more actionable framework for subsequent analyses and interventions.

**Table 2:** Model fit for multiple models.

Cluster model	bic	aic	G^2
1	166825.8745	166699.963	9786.17619
2	163982.1129	163722.883	6889.67201
3	162519.6342	162127.086	5360.66962
4	162050.0189	161524.153	4740.9934
5	161917.9136	161258.729	4445.74064
6	161927.7694	161135.267	4280.28902

Bic, ayesian Information Criterion; aic, Akaike Information Criterion.

### 3.3 Description of the identified groups from the latent class analysis

**Fig. 1:**
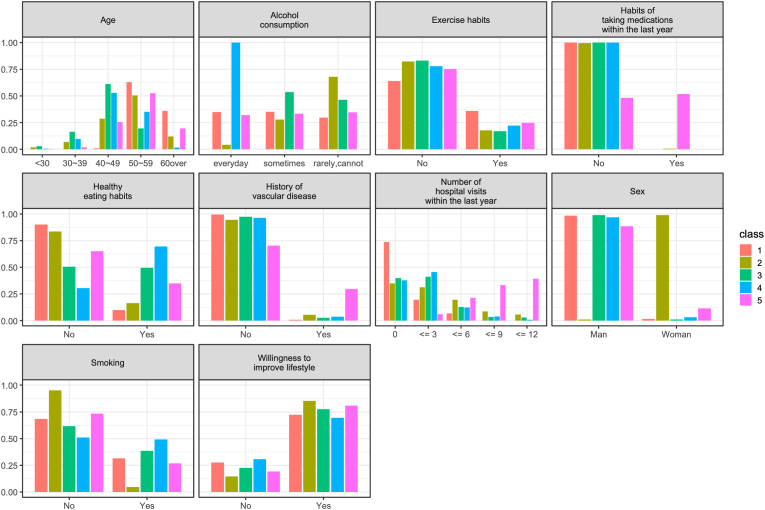
Overview of the distribution in the five latent classes. This figure shows the demographics of the participants in the five classes and the health behavior characteristics of each.

Each cluster was created based on the participants’ demographic and health behavior characteristics. Their clusters are shown in Fig. 1. The characteristics of each of the five clusters are also described in the following.

#### 3.3.1 Cluster 1

Cluster 1 included 2389 participants. They tend to have healthy lifestyles, exercise, and not eat late at night. However, 74% (1749 persons) had not visited a hospital in the previous year, indicating reluctance to visit hospitals. Hence, the label “having healthy lifestyles but disliking hospitals” was used.

#### 3.3.2 Cluster 2

Cluster 2 had 2215 participants. Ninety-five percent (n = 2083) did not smoke, and 68% (n = 1450) drank little or not at all. As in Cluster 1, the frequency of having dinner late in the evening was low. This group was the only one with a high percentage of women: 99% (4350). Therefore, this group was labeled “women with healthy lifestyle behaviors.”

#### 3.3.3 Cluster 3

Cluster 3 had the largest number of participants (4,395). They were generally characterized by relative youth, infrequent drinking, and infrequent hospital visits. Therefore, this group was labeled “general population.”

#### 3.3.4 Cluster 4

Cluster 4 had 2098 participants. They were middle-aged, drank alcohol daily, and had dinner frequently at night and half of them, 1026 (49%), smoked. On the basis of these characteristics, we named this group the “middle-aged group in need of lifestyle improvement.”

#### 3.3.5 Cluster 5

Cluster 5 consisted of 1071 participants, of whom 774 (73%) were 50 years of age or older (“the older age group”). The percentage of participants taking lifestyle-related medications was as high as 53% (554), and the percentage of participants with an actual history of lifestyle-related diseases was also high at 30% (318). In other words, this group was an older group with more frequent hospital visits and lifestyle-related diseases. Hence, this group was labeled “a group of treatment for lifestyle-related diseases.”

## 4 Discussion

The study was conducted as clustering using LCA based on health behavior characteristics taking into account the age and gender of individuals at risk of NCDs in the Japanese working population. The identification of these clusters provides a more nuanced understanding of the health behaviors of the working population in Japan. Each cluster has unique health behaviors and challenges. Cluster 1, labeled as “having healthy lifestyles but disliking hospitals,” highlights a group that maintains healthy behaviors. However, their aversion to hospitals suggests they might be missing out on regular check-ups or necessary medical attention. This reveals a potential gap in the healthcare system. For this group, interventions could emphasize the importance of regular medical check-ups and address any barriers or misconceptions about hospital visits. In cluster 2, which consists mainly of “women with healthy lifestyles”, the data suggest that gender-specific interventions could be particularly effective. Health promotion campaigns tailored specifically to women, emphasizing the benefits of maintaining healthy behaviors, could be a strategic approach. The “general population” represented by Cluster 3 was the largest segment. This highlights the importance of broad public health interventions. However, the overarching nature of this cluster also indicates a potential need for further research. Identifying subgroups within this cluster could lead to more targeted interventions. Cluster 4, described as the “middle-aged group in need of lifestyle improvement,” identifies behaviors such as excessive alcohol consumption and late-night dinners as potential areas of concern. Health campaigns that target middle-aged individuals, highlighting the risks of these behaviors and suggesting healthier alternatives, may resonate well with this group. Lastly, Cluster 5, known as “a group of treatment for lifestyle-related diseases,” comprises an older demographic with a higher prevalence of lifestyle-related diseases. This calls for interventions that focus on disease management and prevention. Strategies might include programs that promote regular medical check-ups, adherence to medication schedules, and lifestyle changes to manage and prevent further health complications.

The clustering was at the extremes, with clusters 1 and 2 being healthy clusters and clusters 4 and 5 represent unhealthy features.

Healthy clusters were observed in studies covering a variety of countries (de Vries et al., 2008; Poortinga, 2007; Schneider et al., 2009). The formation of clusters of healthy people was typical in various population samples. Consider clusters 4 and 5, which have multiple unhealthy behaviors. This suggests that unhealthy behaviors tend to overlap. For example, a person with a drinking habit is likely to have an unhealthy diet. This clustering of multiple unhealthy behaviors was also observed in studies of other countries (Poortinga, 2007; Schuit et al., 2002).

One of the characteristics that separates the healthy groups, Cluster 1 and Cluster 2, is “the number of visits to a hospital.” “The number of visits to a hospital” was also the key variable separating 4 and 5. Furthermore, the variable “the number of visits to a hospital” distinguishes Cluster 1 from Cluster 5. These results suggest that “the number of visits to a hospital” may be one of the key health behavior variables in creating clusters for this study's sample. One study reported that the frequency of hospital visits tended to be higher among older people (Savageau et al., 2006). Cluster 5 was the older age group, which suggests that age may be related to the frequency of hospital visits. Another interesting study highlighted the efficacy of screening for high blood pressure in dental health care, emphasizing the importance of regular medical check-ups in detecting previously unknown health conditions (Engström et al., 2011).

Gender played a significant role in cluster formation, as seen with the “women with healthy lifestyle behaviors” in Cluster 2. Our findings align with the broader literature suggesting gender differences in health behaviors. In the context of our study, a research from the United Kingdom indicated that men were more likely to form unhealthy clusters compared to women (Poortinga, 2007). A study of retirees in Taiwan revealed gender differences in health-related behaviors, with female retirees being less likely to consume tobacco, alcohol, and betel nuts and more likely to avoid high-fat and pickled foods compared to males (Huang et al., 2019). Further, gender has been shown to influence perceptions of and adherence to health-related behaviors after major medical interventions, such as heart transplantation (Rose Epstein et al., 2022).

On the other hand, age was not a major factor in the formation of health behavior clusters in our study. We found both young and old people in healthy and unhealthy clusters. This finding was similar to other studies that showed that age was not always the main factor in health behavior clusters (Meader et al., 2016; Poortinga, 2007). For example, Olson et al. found that gender was more important than age in clusters of health behaviors among young adults in the U.S (Olson et al., 2017). Another study by Rabel et al. investigated health behaviors including drinking, eating, and exercise and found that age was only one of many factors (Rabel et al., 2018). Thus, while age can influence health behaviors, its role in clustering may vary depending on the group and behaviors being studied.

The previous study also concluded that alcohol consumption and smoking were important factors in forming the clusters (Meader et al., 2016). Alcohol consumption and smoking were also factors in this study as variables that classified clusters 2 and 4. Similar results were found in many countries, such as the Netherlands, Hong Kong, South Korea, Ireland, and Australia (Chou, 2008; Conry et al., 2011; Griffin et al., 2014; Kang et al., 2010; Schuit et al., 2002). The findings of the current study, which included a different population, would further reinforce the importance of alcohol consumption and smoking as factors in forming the clusters. In addition, the habit of exercising at least twice a week for at least 30 min was a variable that distinguished Cluster 1. Exercise, as underscored by numerous studies, plays a pivotal role in preventing or ameliorating NCDs (Gaesser, 2007; Griffin et al., 2014; Meader et al., 2016; Noble et al., 2015). In many studies, exercise habits emerge as a key health behavior. For instance, a study found that the average number of health-promoting lifestyles among subjects was of medium-low level, emphasizing the need for improvement in areas such as health responsibility, exercise, and nutrition (Wen et al., 2019). However, in our study, exercise did not have a strong influence on clustering. This attenuation might be attributable to the pronounced influence of other behaviors, notably alcohol consumption and smoking. These behaviors can have immediate health effects and might be more prevalent among the participants. It's possible that in the Japanese population of working age, certain types of behavior are more dominant than physical activity. For instance, dietary patterns, especially those involving low grain intake, might play a significant role in influencing behaviors (Toyomaki et al., 2017). This study on middle-aged Japanese individuals found a relationship between low grain intake dietary patterns and impulsive behaviors, suggesting that such dietary habits could be more prevalent and influential in this demographic than exercise. Smoking and alcohol consumption, in particular, can be more defining for clustering than exercise. Further research is needed to understand how these behaviors interact and influence health.

### 4.1 Limitations

The study has several limitations. First, respondents may unconsciously provide socially desirable responses, and reliance on self-reported health behaviors introduces potential bias. The use of dichotomous or ordinal scales for health behavior variables may also introduce inaccuracies in comparison to more detailed scales or open-ended descriptions (MacCallum et al., 2002). Another limitation was the limited number of health behavior variables. While the study strategically focused on certain variables, it acknowledges the limitation of not including some health behavior variables like educational background and nutritional status (Schuit et al., 2002).

However, despite these limitations, the study possesses advantages that outweigh them. Given the large sample size of this study, the design was intentionally kept simple for manageability and clarity. The questionnaire used in this study was a standard tool employed nationally, ensuring its reliability and making it representative of the general Japanese population. This standardized questionnaire is designed to be straightforward, minimizing the potential for bias in responses. By utilizing a simple and standardized questionnaire, we aimed to reduce the potential biases and ensure consistent data collection across the vast number of participants. The health behavior variables selected for this study have been recognized as important factors in various international studies. By focussing on these universally accepted variables, this study not only maintains a clear focus, but also facilitates cross-national comparisons. This approach enhances both the applicability of our research and the generalizability of the findings, making a significant contribution to the broader field of health behavior research. The study focuses on the health behavior of a large sample of more than 10,000 working-age Japanese. It is further focused on the population that needed care for NCDs. Virtually no studies meet these criteria. Another important feature of the analysis is that it was performed using LCA. This allowed for a more flexible and distinctive cluster classification. The study's results suggest the need for interventions tailored to these characteristics in a population approach to public health. They also provide valuable information when recommending health consultations to prevent severe disease.

### 4.2 Practical implications

Understanding the characteristics of each cluster from this study may enable health professionals and policy makers to tailor interventions and health promotion strategies to the specific needs of each group. Our study elucidate the health behaviors of the Japanese working population and offers insights for targeted interventions. Cluster 1's reluctance towards hospitals suggests the viability of alternative healthcare approaches, such as telemedicine or community-based programs, ensuring care without the traditional hospital setting's constraints. A study from four European countries indicated a growing interest in remote monitoring for managing chronic diseases (Rojahn et al., 2016). This approach might be especially beneficial for those hesitant about traditional hospital visits, suggesting a promising direction for future healthcare strategies. Cluster 2, primarily consisting of health-conscious women, highlights the potential of gender-specific health strategies. For instance, interventions addressing pregnancy and reproductive health concerns might be particularly beneficial for this group. There is potential benefit in using technology-mediated interventions for cluster 4, which includes middle-aged individuals with suboptimal lifestyles. Wearable health devices, for example, have been shown to be an aid to lifestyle modification, particularly in individuals with risk factors for metabolic syndrome. A study from South Korea highlighted the habitual use of these devices in promoting sustained healthy behaviors among middle-aged individuals, underscoring the importance of aligning user expectations with device performance for effective self-health management (Ha et al., 2023). Cluster 5, characterized by older age and frequent hospital interactions, underscores the need for enhanced medical care. Chronic disease management is critical for this group. Ageing is often associated with multiple chronic conditions that affect quality of life (Han et al., 2022). Implementation of health status feedback systems and tailored care interventions may reduce risks and improve well-being in older adults. Incorporating these insights into healthcare initiatives promises more tailored interventions, aiming to elevate health outcomes for the varied segments of the Japanese working demographic.

## 5 Conclusion

In conclusion, this study reveals more discernible patterns in health behavior in a population sample with extensive real-world data in Japan. Knowledge of clusters common to large populations is important information for public health policies to improve health. The study results revealed that five clusters based on health behaviors form among the population of those in need of treatment for NCDs. This result suggested the need for an approach tailored to each group's characteristics, rather than treating them as a uniform group, when considering population approaches in public health.

## Future research

Building on the findings of this study, it would be valuable to design and implement interventions tailored to each identified cluster. Understanding the unique characteristics of each group can guide the development of targeted strategies to promote health behavior change. Evaluating the effectiveness of these tailored interventions will further advance our understanding and ensure that health initiatives are tailored to meet the specific needs of diverse populations. In addition, exploring the long-term effects of health behaviors within each cluster and tracking health outcomes over time may illuminate the lasting effects of these behaviors. The integration of technological tools, such as wearable devices, can improve data accuracy and pave the way for timely interventions.

## Ethical statement

Since the study data were provided anonymously, and the study participants did not receive any intervention, informed consent for study participation was waived. As the study using anonymized data is outside the scope of the national guidelines “Ethical Guidelines for Medical and Health Research Involving Human Subjects”, the ethical committee review and individual-level consent were not required. However, the study protocol was reviewed and approved by the Institute of Transdisciplinary Sciences Ethics Committee, Kanazawa University. Any potential risks were minimized, and participants' confidentiality and anonymity were maintained throughout the study. Additionally, the survey was conducted in consideration of the Declaration of Helsinki (revised October 2013) by the World Medical Association and the Ethical Guidelines for Medical Research Involving Human Subjects. The data obtained from the study are stored securely and will be used solely for the purpose of this research.

## Availability of data and materials

The data used in this study is the property of PREVENT Co., Ltd. and the purpose of use is restricted by PREVENT's policy on data utilization. The data used in this study was also used under contract and is not available to the public.

## Funding

Not applicable.

## Authors’ contributions

All authors contributed significantly to this article. TM and YH conceived and designed the study; TM, MK and KY analyzed the data and wrote the manuscript. MK, KT, MI and TH collected the data. MK and YH supervised the implementation; All authors provided critical comments on the manuscript and revised the manuscript. All authors read and approved the final manuscript.

## Consent for publication

Not applicable.

## Declaration of competing interest

MK. is a non-regular staff member of PREVENT Inc. YH is a founder and stockholder of PREVENT Inc. TM, KT, KY and MI are employees of PREVENT Inc.

## Data Availability

Data will be made available on request.

## Abbreviations

NCDs: Noncommunicable Diseases
LDL: Low-Density Lipoprotein
HDL: High-Density Lipoprotein
LCA: The latent class analysis
BIC: The Bayesian Information Criterion
AIC: Akaike Information Criterion
